# Optimization and Validation of Indirect ELISA Using Truncated TssB Protein for the Serodiagnosis of Glanders amongst Equines

**DOI:** 10.1155/2014/469407

**Published:** 2014-02-03

**Authors:** Harisankar Singha, Praveen Malik, Sachin K. Goyal, Sandip K. Khurana, Chiranjay Mukhopadhyay, Vandana K. Eshwara, Raj K. Singh

**Affiliations:** ^1^National Research Centre on Equines, Sirsa Road, Hisar, Haryana 125 001, India; ^2^Veterinary Type Culture Collection, National Research Centre on Equines, Sirsa Road, Hisar, Haryana 125 001, India; ^3^Department of Microbiology, Kasturba Medical College, Manipal University, Manipal, Karnataka 576104, India

## Abstract

*Objective*. To express truncated TssB protein of *Burkholderia mallei* and to evaluate its diagnostic efficacy for serological detection of glanders among equines. *Materials and Methods*. In an attempt to develop recombinant protein based enzyme-linked immunosorbent assay (ELISA), N-terminal 200 amino acid sequences of *B. mallei* TssB protein—a type 6 secretory effector protein—were expressed in prokaryotic expression system. Diagnostic potential of recombinant TssB protein was evaluated in indirect ELISA using a panel of glanders positive (*n* = 49), negative (*n* = 30), and field serum samples (*n* = 1811). Cross-reactivity of the assay was assessed with equine disease control serum and human melioidosis positive serum. *Results*. In comparison to CFT, diagnostic sensitivity and specificity of ELISA were 99.7% and 100%, respectively. *Conclusions*. The indirect ELISA method using the truncated TssB offered safer and more rapid and efficient means of serodiagnosis of glanders in equines. These data highlight the use of TssB as potential diagnostic antigen for serological diagnosis of glanders.

## 1. Introduction

Glanders is a fatal infectious disease mainly of horses, donkeys, and mules and also communicable to man. The disease is caused by the Gram-negative nonmotile bacillus *Burkholderia mallei*, a close relative of *Burkholderia pseudomallei*—the causal agent of melioidosis in humans. The glanders in equines is characterized by nodular lesions of the lungs and other organs as well as ulcerative lesions of the skin and mucous membranes of the nasal cavity and respiratory passages [1]. Horses are the primary carriers of disease and are largely responsible for transmitting the infection to healthy animals and humans [2]. Lack of prophylactic vaccine together with challenging treatment regime renders *B. mallei* a pathogen of choice for potential biological weapon.

Isolation and identification of the organism from fresh clinical specimens (cutaneous lesions and nasal exudates) is the “gold standard” for diagnosis of glanders. However, clinical and bacteriological identification of causative agent from equines during the early stages of infection or in clinically inapparent cases is not always attainable. Although complement fixation test (CFT) and mallein test are officially recognized by OIE for international trade of equidae [3], species specific biasness of the test and need of technical expertise are the major limitations of these assays. Different ELISA formats including avidin-biotin dot ELISA [4], cELISA [5], and indirect ELISA [6] have been developed for diagnosis of glanders. However, these assays either use crude or purified bacterial fractions as antigens thereby affect the test specificity.

The type six secretion system (T6SS) is conserved in numerous Gram-negative pathogens and symbionts that interact closely with eukaryotic cells [7, 8]. The *B. mallei* T6SS gene cluster 1 (BMAA0730 to BMAA0744) and homologous gene in *B. pseudomallei* (BPSS1496–1512) are important for actin-based motility, multinucleated giant cell (MNGC) formation, intracellular growth in murine macrophages, and virulence in hamsters [7, 9]. The TssB protein encoded by BMAA0743 locus of *B. mallei* T6SS-1 is a secretory effector-like protein and thus the TssB protein may be used as diagnostic antigen for serological detection of glanders. Therefore, the objectives of present study were to express immunodominant epitope of TssB protein of *B. mallei* and to evaluate its diagnostic efficacy for serological detection of glanders among equines.

## 2. Materials and Methods

### 2.1. Customization and Synthesis of *tss*B Gene

The 1500 bp sequences of *tss*B gene of *B. mallei* (locus tag BMAA0743) were retrieved from National Center for Biotechnology Information (NCBI) database and antigenicity plot of the translated sequence was determined by Kolaskar and Tongaonkar method [10] using CLC work bench v 6.1 (http://www.clcbio.com/). Based on antigenicity index, first 600 bases of the *tss*B gene encoding N-terminal 200 amino acid sequences were selected for expression. The codons of the selected sequences were optimized according to codon usage in *E. coli* K-12 strain. The *Bam*HI and *Hind*III restriction sites were placed at 5′ and 3′ end of the sequences, respectively, for ease of downstream application. The customized *tss*B gene was commercially synthesized in pUC57 vector (BioBasic Inc., Canada).

### 2.2. Production of Recombinant TssB Protein

The gel purified *Bam*HI and *Hind*III restricted 600 bp *tss*B gene was directionally ligated to pQE30 expression vector (Qiagen, Germany) and transformed into chemically competent *E. coli* M15 cells. Positive clones expressing recombinant TssB protein were screened by sodium dodecyl sulphate polyacrylamide gel electrophoresis (SDS-PAGE). Single clone showing maximum expression of TssB protein after 4 h of induction by 1 mM IPTG (isopropyl-D-thiogalactopyranoside) was selected for production of recombinant protein. The N-terminal histidine-tagged TssB protein was purified by Ni^+^-NTA agarose (Qiagen, Germany) as per manufacturer's instruction. Eluates containing recombinant TssB protein were pooled and dialyzed against 0.01 M phosphate-buffered saline (PBS; pH 7.2). The protein concentration was determined using a bicinchoninic acid (BCA) protein assay kit (Merck Millipore, India). Protein in 1 mL aliquots of 0.5 mg/mL concentration was kept in −20°C for future use.

### 2.3. Serum Panel

Diagnostic performance of the recombinant TssB protein was evaluated using a panel of glanders positive and negative equine serum samples maintained in the serum repository of National Research Centre on Equines (NRCE), Hisar, India. A total of 49 glanders positive equine serum samples collected during glanders outbreak between 2006 and 2011 were used as positive control [11]. Negative control consists of 40 serum samples of healthy equines which did not show any clinical signs of glanders and were consistently negative by CFT. The cross-reactivities of recombinant TssB protein were assessed with melioidosis positive human serum. Serum samples from human patient suffering from melioidosis (*n* = 6) and septicemia caused by other infectious bacteria (*n* = 10) were used as melioidosis positive control and disease control serum, respectively. The human serum samples were collected at Kasturba Medical Hospital, Manipal, Karnataka, India. The serum panel also contained 14 samples from equine infected with bacteria like *Streptococcus equi* subspecies *equi* (*n* = 5), *Streptococcus equi* subspecies *zooepidemicus* (*n* = 5), and *Pseudomonas aeruginosa* (*n* = 5) for determination of the specificity of the ELISA. Equine serum samples (*n* = 1811) received from different race clubs and private farms or collected from field for serosurveillance of equine disease were used as test samples for diagnostic evaluation of the indirect ELISA.

### 2.4. Determination of TssB Specific Antibody by Western Blot

Specific reactivity of recombinant TssB protein to *B. mallei* antibody was determined by western blot. Approximately, 600 ng TssB protein/well was transferred to PVDF membrane. The unbound surface of the membrane was blocked by PBS containing 10% skimmed milk for overnight at 4°C. The membrane was cut into strips, washed with PBS containing 0.05% Tween 20 (PBS-T), and incubated with 1 : 200 dilution of serum for 2 h at 37°C. The strips were washed with PBS-T and incubated with 1 : 10000 dilutions of HRP conjugated rabbit anti-horse or anti-human IgG antibody as per need (Sigma-Aldrich, USA) for 1 h at 37°C. The reaction was developed with diaminobenzidine (6 mg/10 mL 50 mM Tris buffer pH 7.6) and 30 *μ*L of 30% H_2_O_2_. The reaction was stopped by washing the membrane in distilled water and results were recorded.

### 2.5. Optimization of Indirect ELISA

The optimal concentration of ELISA reagents (TssB protein, serum, and secondary antibody dilution) was determined by checkerboard titration method using known glanders positive and negative equine serum. The optimal ELISA reagents concentration was assumed to be for those showing the highest discrimination between positive and negative serum. After optimization of the assay, 1811 equine serum samples were assayed by ELISA. Pool of four positive and four negative serum samples were included in each ELISA plate along with field serum samples for monitoring the accuracy of the assay.

In brief, 96-well microtiter plates (Greiner Bio One, USA) were coated with TssB protein for overnight at 4°C followed by washing with PBS-T and blocking with 6% skimmed milk in PBS-T for 1 h at 37°C. Serum samples were diluted in dilution buffer (2% (w/v) skimmed milk in PBS-T) and 100 *μ*L of diluted serum was added in each well. The plates were incubated for 1 h at 37°C and washed five times with PBS-T. The diluted anti-horse conjugate (100 *μ*L) was added in each well and incubated for 1 h at 37°C. Following washing steps, 100 *μ*L of substrate solution containing 200 *μ*mol of orthophenylene-diamine in citrate perborate buffer (Sigma-Aldrich, USA) was added in each well and plate was kept in dark at 37°C for 10 min. Finally, the reaction was stopped by addition of 50 *μ*L of 1 M H_2_SO_4_ and the absorbance was read at 492 nm in a microtiter plate reader (Titertek Multiskan, Finland).

### 2.6. Complement Fixation Test (CFT)

ELISA results were compared with CFT which was performed as per standard protocol described by the Office International des Épizooties (OIE) [3] using commercial kits (Bioveta, a.s., Czech Republic) for detection of *B. mallei* antibodies. The CFT titer of 1 : 8 and above was considered positive for glanders.

### 2.7. Data Analysis

The optimum cutoff point of ELISA was determined by receiver operative curves (ROC) analysis using normalized OD_492_ value (PP%) as described elsewhere [12, 13]. For determination of the cutoff point, absorbance data of ELISA (O.D. values) were analyzed using Windows 7 Microsoft Excel programme (Microsoft Corp, Redmond, WA) and were normalized using the following formula:

(1) Percent  positivity  (PP%) =(OD492  sample  serum−OD492  negative  control)(OD492  positive  control−OD492  negative  control)  ×100%.

Test samples with PP% value greater than the cutoff point were treated as positive. Relative sensitivity and specificity of the assay were determined by following calculation and the results were expressed as percentages:

(2) $$\begin{array}{c} \text{Sensitivity}=\frac{\text{True}\;\text{positive}}{\left(\text{True}\;\text{positive}+\text{False}\;\text{negative}\right)}\\ \text{Specificity}=\frac{\text{True}\;\text{negative}}{\left(\text{True}\;\text{negative}+\text{False}\;\text{positive}\right)}. \end{array}$$

## 3. Result

### 3.1. Expression and Purification of Recombinant TssB Protein

**Figure 1:**
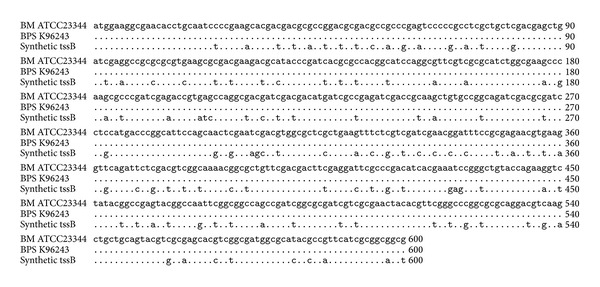
Sequence alignment of 5′ terminal 600 bp *tss*B gene of *B. mallei*, *B. pseudomallei,* and customized *tss*B gene. Identical sequences are represented by dot. Nonidentical sequences at third base of codon have been modified during the process of codon optimization.

**Figure 2:**
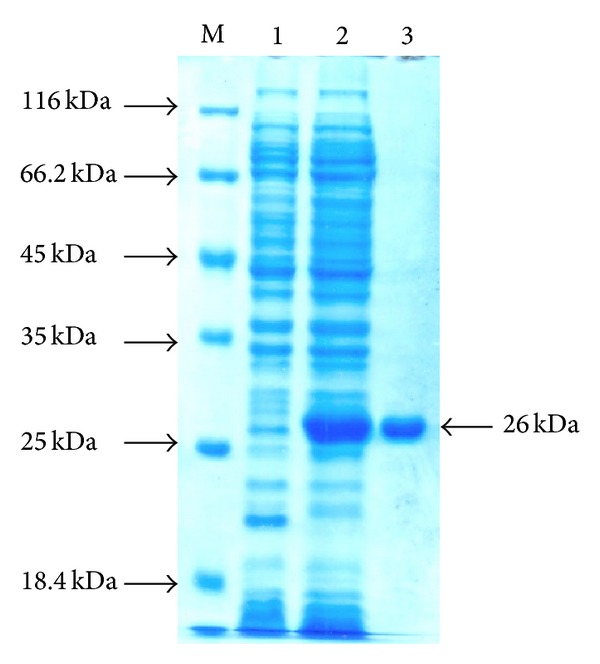
SDS-PAGE analysis of recombinant TssB protein. Lane M: protein molecular weight marker (Fermentas, number SM0431), Lane 1: uninduced control, 2: IPTG induced cell lysate, and 3: Nickel-NTA affinity column purified protein.

The codon optimized 600 bp immunodominant fragment of *tss*B gene is shown in Figure 1. To reduce the G + C content of the targeted gene fragment, only third base of the codons was changed which results in changes in DNA sequence but the amino acid sequence remains unaltered. Besides *B. mallei*, the deduced amino acid sequence of truncated TssB also showed 100% identity with the protein homologue present in *Burkholderia pseudomallei* (data not shown). The recombinant *E. coli* carrying pQE30: *tss*B cassette expressed 26 kDa proteins upon IPTG induction (Figure 2 Lane 2). SDS-PAGE analysis of eluted fractions revealed that protein was purified to homogeneity and no contaminating protein band was found.

### 3.2. Western Blot Analysis

**Figure 3:**
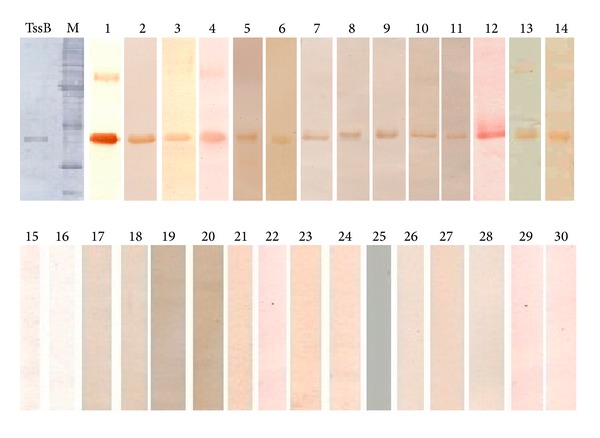
Specific immune reactivity of recombinant TssB protein in western blot. Lanes TssB and M: amido black stained recombinant TssB protein and protein molecular weight marker, respectively, upon transfer on PVDF membrane. Lanes 1–14: Glanders positive equine serum, Lanes: 15–18: negative control serum, Lanes 19–22: melioidosis positive human serum. Equine disease control serum: Lanes 23–25: *Streptococcus equi* infection (strangles); Lane 26–28: *Streptococcus zooepidemicus* infection; Lane 29-30: and *Pseudomonas aeruginosa* infection.

In western blot, glanders positive equine serum gave a positive reaction band at 26 kDa position. Negative control serum, melioidosis (human), and other equine disease control serums showed no reactivity with TssB protein (Figure 3).

### 3.3. Diagnostic Efficacy of Indirect ELISA

**Figure 4:**
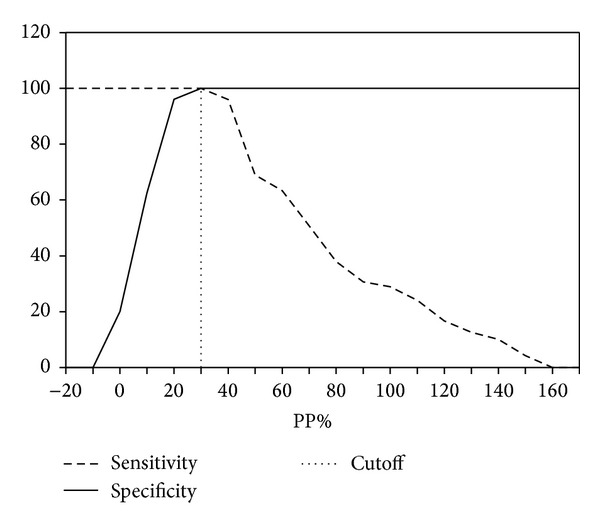
Determination of cutoff point of indirect enzyme-linked immunosorbent assay.

**Figure 5:**
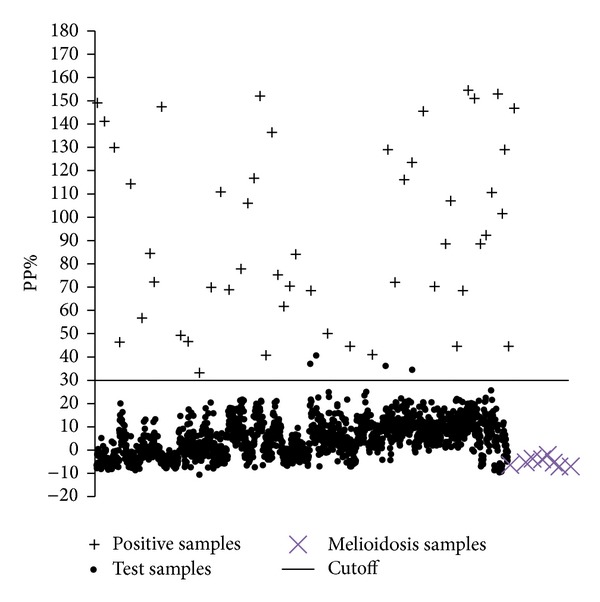
Distribution of PP% value of positive and negative serum samples. The horizontal line represents the cutoff value [percent positivity (PP% = 30)]. Glanders positive serum is indicated by + sign, dot represents the test serum samples, and melioidosis positive human serum is indicated by × symbol.

Optimal concentration of ELISA reagents was found as follows: 150 ng of purified TssB protein/well, 1/200 serum dilution, and 1/15000 conjugate dilution. The percent positivity percentage (PP%) value of 30.0 was calculated as cutoff point (Figure 4); serum samples showing PP% greater than or equal to 30.0 and less than that were regarded as positive and negative, respectively. Out of 1811 equine serum samples (*n* = 1500 horses, *n* = 150 donkeys, and *n* = 161 mules), 4 samples were found false positive in ELISA (Figure 5). However, there were none that were negative in ELISA but positive in the CFT. The PP value of melioidosis and other equine disease control serum samples was below the cutoff. The relative specificity and sensitivity of the ELISA were 99.7% and 100%, respectively.

## 4. Discussion

Glanders is a notifiable disease in India and presently follows the “test and stamp-out policy” as the only control measure. The OIE approved mallein test and complement fixation test (CFT)—commonly used for the diagnosis of glanders—suffer from lack of sensitivity particularly in clinically advanced cases [14, 15], and additionally mallein test causes transient seroconversions in healthy uninfected animals [16]. The CFT for glanders is so far the only officially recognized serological laboratory test for international trade of equidae and remains the preferred diagnostic tool in eradication programme. However, complement fixation test (CFT) has drawback of producing notable number of nonspecific results especially with donkeys and mules serum [14, 17].

Partially purified or crude antigen preparations of *B. mallei* were evaluated in Rose Bengal plate agglutination test (RBT) [15], western blot assays [17], a competitive ELISA [5], and indirect ELISA [6]. The presence of cross-reacting epitopes in antigen preparation affects the specificity of these assays. Further, requirement of strict biosafety measures and the possible laboratory-acquired infection of *B. mallei* during the preparation of antigens is still a serious drawback. Although many PCR techniques for detection of *B. mallei* DNA have been described [18, 19], specificity of PCR detection method has not been tested in clinical samples so far. Moreover, PCR techniques have got limited acceptance in surveillance of equine populations, owing to the need for containment laboratory facilities, trained personnel, and financial resources.

Previously, Defence Research and Development Establishment (DRDE), Gwalior, and our lab in collaborative research project have reported the recombinant protein based ELISA for glanders diagnosis [20, 21]. In a continuous effort, we describe here the homogeneous production of truncated TssB protein of *B. mallei* and evaluated the diagnostic potentiality of the recombinant TssB protein in indirect ELISA. Cross-reactivity experiment with human melioidosis serum and equine disease control serum showed that the TssB protein is highly specific to detect *B. mallei* antibodies. In view of the high percentage of sequence identity of *B. mallei* TssB protein with *B. pseudomallei* protein homologue, nonreactivity of melioidosis positive serum with TssB observed in this study is really surprising. The similar findings were previously observed by Pal et al. [21] where *B. mallei* hypothetical protein BMA10229_0375 did not cross-react with melioidosis serum despite showing considerable sequence identity (~91%) with *B. pseudomallei* homologue proteins. Nonreactivity of TssB protein with melioidosis serum of Indian origin may be substantiated by two facts: (i) genetic diversity of Indian *B. pseudomallei* isolates from other strains originated from melioidosis endemic region like Thailand and Australia [22] and (ii) differential regulation and function of TssB in *B. mallei* and *B. pseudomallei* infection [9, 23]. Therefore, on the basis of information obtained from literature and findings of the present experiment, it appears that expression and secretion pattern of TssB may be different in clinical infection of *B. mallei* and *B. pseudomallei* which may lead to variation in antibody production against the TssB protein.

The indirect ELISA method using recombinant TssB protein offered safer and more rapid and efficient means of serodiagnosis of glanders in equines. Further validation of the assay with equine serum from glanders endemic and glanders-free countries will be useful for wider acceptance of the TssB protein based ELISA.
